# West Nile Virus in the United States — A Historical Perspective

**DOI:** 10.3390/v5123088

**Published:** 2013-12-10

**Authors:** John T. Roehrig

**Affiliations:** Arboviral Diseases Branch, Division of Vector-Borne Diseases, National Center for Zoonotic and Emerging Infectious Diseases, U.S. Centers for Disease Control and Prevention, 3156 Rampart Road, Fort Collins, CO 80521, USA; E-Mail: jtr1@cdc.gov; Tel.: +1-970-221-6442; Fax:+1-970-494-6631

**Keywords:** West Nile, New York City, U.S. outbreak, zoonotic viruses

## Abstract

Prior to 1999, West Nile virus (WNV) was a bit player in the screenplay of global vector-borne viral diseases. First discovered in the West Nile District of Uganda in 1937, this *Culex* sp.-transmitted virus was known for causing small human febrile outbreaks in Africa and the Middle East. Prior to 1995, the last major human WNV outbreak was in the 1950s in Israel. The epidemiology and ecology of WNV began to change in the mid-1990s when an epidemic of human encephalitis occurred in Romania. The introduction of WNV into Eastern Europe was readily explained by bird migration between Africa and Europe. The movement of WNV from Africa to Europe could not, however, predict its surprising jump across the Atlantic Ocean to New York City and the surrounding areas of the United States (U.S.). This movement of WNV from the Eastern to Western Hemisphere in 1999, and its subsequent dissemination throughout two continents in less than ten years is widely recognized as one of the most significant events in arbovirology during the last two centuries. This paper documents the early events of the introduction into and the spread of WNV in the Western Hemisphere.

## 1. Introduction

There are over 500 registered arthropod-borne viruses (arboviruses). Arboviruses are composed of virus members of the Flaviviridae (of which West Nile virus, WNV, is one), Togaviridae, Bunyaviridae, Rhabdoviridae, Reoviridae, and Orthomyxoviridae families. While some of these viruses (e.g., dengue virus, DENV) have global distribution, many have distinct geographic ranges. For example, yellow fever virus (YFV) is essentially a virus of equatorial Africa and Central and South America, even though the YFV mosquito vector—*Aedes (Ae.) aegypti*—can be found throughout the world. The epidemic encephalitic alphaviruses—eastern, western, and Venezuelan equine encephalitis viruses (EEEV, WEEV, and VEEV)—are essentially Western Hemisphere viruses. The arthrogenic alphaviruses (e.g., O’nyong nyong virus, ONNV, Chikungunya virus, CHIKV, and Ross River virus, RRV) are limited to the Eastern Hemisphere and Australia. Much of these geographic limitations are due to the complex arboviral life cycles that require specific reservoir- and amplifying-hosts, endemic-, epidemic-, and bridge-vectors, represented in unique ecological habitats.

## 2. Status of Vector-Borne Encephalitides in 1999 in the United States

Epidemic arboviruses in the United States (U.S.) during the late 1900s were relatively non-existent [1]. The last major human epidemic of St. Louis encephalitis virus (SLEV) occurred in the mid to late 1970s, although smaller outbreaks of SLEV continued to occur periodically throughout the U.S. after that [2,3,4,5,6]. SLEV and WNV are members of the Japanese encephalitis virus (JEV) serocomplex and all three viruses are very closely related both genetically and antigenically [7,8,9]. While EEEV caused occasional infections in horses, emus, and humans, these outbreaks were very small and limited to areas where the ecosystem supported the EEEV transmission cycle [10]. Major epidemics- and even smaller outbreaks- of WEEV had stopped altogether [11]. The most important cause of mosquito-borne viral encephalitis in humans in the USA at this time was LaCrosse virus (LACV) [11]. The geographic range of this bunyavirus in the U.S. included the mid-Atlantic states, upper Midwest, and Southwest Louisiana. The LACV is transmitted by the mosquito *Ae. triseriatus*, so its distribution was limited to well-defined geographic niches containing hardwood forests that supported mosquito breeding in treeholes. Because of this dearth of arboviral activity in the U.S., both federal and state laboratories responsible for arboviral diagnosis, prevention, and control struggled to remain in existence. 

## 3. Diagnosis of Arboviral Disease

A variety of arboviruses cause human encephalitis. Diagnosis of human infection is not easy, and is based upon: (1) the presence or absence of antiviral IgM (as measured in IgM antibody capture-ELISA, MAC-ELISA) in acute-phase serum or cerebrospinal fluid (CSF) specimens; (2) a virus-specific four-fold or higher IgG titer rise or fall from acute- to convalescent-phase paired serum specimens; and/or (3) direct demonstration of infectious virus, viral antigen, or viral RNA in serum, CSF, or tissue specimens. Specimens must be obtained from an individual with symptoms clinically compatible with encephalitis. Serodiagnosis of human flaviviral encephalitis is even more problematic because of the close antigenic relationships among all flaviviruses. Because the flavivirus envelope (E) protein expresses epitopes that are shared by all flaviviruses, an individual infected with SLEV, for example, produces antiviral antibody that cross-reacts with other flaviviruses as distantly related as DENV and YFV. Quite significant serological cross-reactions are observed among virus members of the same serocomplex (e.g., SLEV, WNV and JEV) [12,13] (Table 1). Differentiation of human SLEV, WNV, and JEV infections require use of more sophisticated virus-specific serological tests such as the plaque-reduction neutralization test (PRNT). Of course these infections are more easily diagnosed if virus or pieces of virus are available for analysis. Flaviviral antigens can be differentiated in a simple immunofluorescence assay using virus-infected cells and virus-specific monoclonal antibodies (MAbs) [14]. Viral nucleic acid can be positively identified in polymerase chain reaction (PCR) assays using primers that amplify RNA from only one virus.

**Table 1:** IgM antibody capture ELISA (MAC-ELISA) flaviviral cross-reactivity of sera from four confirmed WNV encephalitis cases in NYC in 1999.

Serum	SLEV	JEV	WNV	DENV2	YFV	POWV
1	4.96 ^a^	7.75	16.74	2.45	1.82	1.56
2	4.8	13.77	16.68	4.13	2.14	1.75
3	5.45	9.67	16.08	4.09	1.61	1.44
4	4.76	10.07	17.19	3.32	1.62	1.3
+Control	6.5	8.2	6.34	7.45	3.96	4.5

^a^ Results are reported as positive/negative values (P/N) using a 1:400 serum dilution. Any P/N value ≥ 3.0 are positive. P/N values >2.0 but <3.0 are considered suspect and need further serological confirmation. P/N values <2.0 are considered negative. Abbreviations: SLEV, St. Louis encephalitis virus; JEV, Japanese encephalitis virus; WNV, West Nile virus; DENV2, dengue virus serotype 2; YFV, yellow fever virus; and POWV, Powassan virus.

**Figure 1:**
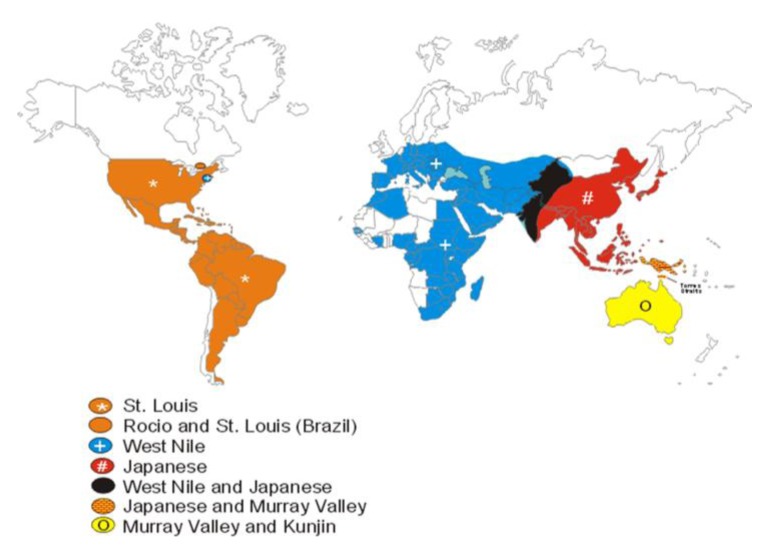
Global distribution of flaviviruses in the JEV serocomplex in 1999.

**Figure 2:**
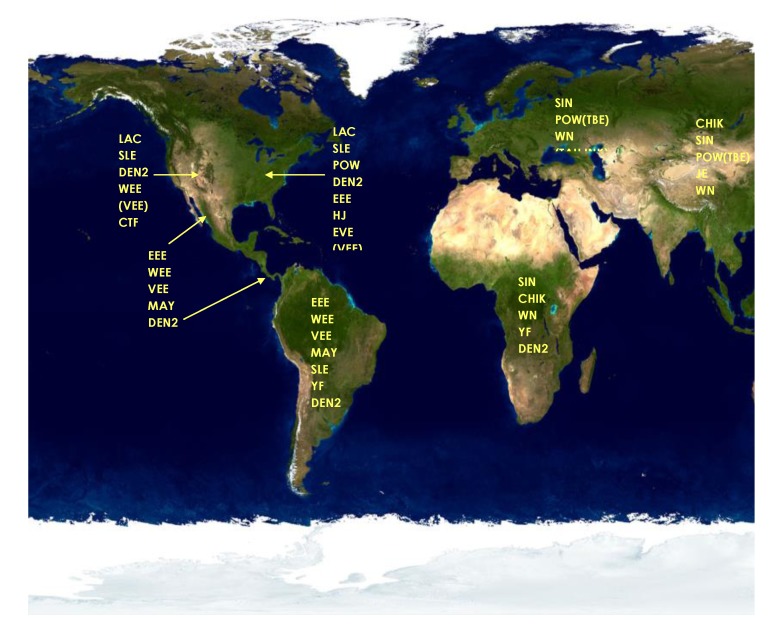
Viral antigen screening panels for arboviral diagnosis used in 1999. Abbreviations: LAC, LaCrosse; SLE, St. Louis encephalitis; WEE, western equine encephalitis; VEE, Venezuelan equine encephalitis; CTF, Colorado Tick fever; DEN2, Dengue 2; POW, Powassan; EEE, eastern equine encephalitis; HJ, Highlands J; EVE, Everglades; MAY, Mayaro; YF, yellow fever; SIN, Sindbis; TBE, tick-borne encephalitis; TAH. Tahanya; INK, Inkoo; CHIK, Chikungunya; JE, Japanese encephalitis; WN, West Nile; SSH, Snowshoe hare; RR, Ross River; BF, Barmah Forest; MVE, Murray Valley encephalitis.

Diagnosticians exploit flaviviral serologic cross-reactivities to their advantage. Of the 70 or so recognized flaviviruses, only a handful of them cause significant human disease, and generally the geographic ranges of the encephalitis-causing viruses are unique (Figure 1). In 1999 the main flaviviruses that caused human encephalitis were SLEV, JEV, Murray Valley encephalitis virus (MVEV), and tick-borne encephalitis virus (TBEV). These viruses were primarily located as follows: Western Hemisphere (SLEV), Southeast Asia and the Indian subcontinent (JEV), Australia (MVEV), and Europe and Asia (TBEV). Less medically important flaviviruses like WNV (Africa and Asia prior to its introduction into North America), Kunjin virus (KUNV, Australia), and Powassan virus (POWV, North America) also had unique geographic distributions. By screening sera using geographically composed panels of arboviruses, the number of antigens needed for testing is greatly reduced. Capitalizing on flaviviral cross-reactivity, unusual flaviviral activity outside of normal geographic ranges can be detected (Figure 2). A caveat of this approach is that a flavivirus arriving in an unexpected region—like WNV in the U.S.—will be detected and identified as a flavivirus serologically, but will not be definitively identified until virus or pieces of virus are available for analysis. This approach to flaviviral diagnostics was in place in 1999 and still is today.

## 4. Outbreak Response to Zoonotic Diseases in the U.S.

The USA Federal response algorithm to zoonotic disease outbreaks is complex, with Departmental responsibilities intersecting in a variety of ways. The CDC is primarily responsible for investigating human disease outbreaks. This overlaps with the Food and Drug Administration’s (FDA) responsibility, if the outbreak is food-borne or associated with a product under FDA jurisdiction. The FDA is also responsible for evaluating and approving commercial diagnostic tests, vaccines, and treatments for human diseases. The U.S. Department of Agriculture (USDA) is responsible for outbreaks in agriculturally important animals, e.g., horses, cattle, pigs, and sheep. The Department of Interior (DOI) is responsible for disease in wild animals. The Department of Defense (DOD) focuses on disease outbreaks in the military. Finally the National Institute of Health (NIH), while having no direct mission in outbreak identification and control, funds the bulk of infectious disease research and some clinical trials in the U.S. This research results in the knowledge and products needed for human health. The Department of Homeland Security was not in existence in 1999, it had no role in the public health response to the WNV epidemic in the U.S.

The responsibility for disease outbreak response goes beyond the federal level. State, county, and local public health organizations function as the front line for identifying disease activity. They are frequently the first responders in terms of disease surveillance, epidemiologic investigations, lab diagnosis, and implementation of control measures. In fact the CDC does not participate in an outbreak investigation without being invited to do so by state public health officials. Because of its national perspective, however, CDC participation is usually required during multi-state disease outbreaks or epidemics. Such was the case in 1999 with the WNV outbreak. Due to dwindling public health funding, the state and local capacity to respond to an arboviral epidemic/epizootic was greatly limited at this time. Only a few larger state public health labs (e.g., Florida, California, and New York) had the infrastructure and expertise in place to mount a response to WNV. Some local jurisdictions that routinely see arboviral activity and maintained comprehensive mosquito abatement districts also had a good infrastructure for mosquito surveillance and control. Fortunately, immediate increased funding by Congress targeted for the WNV response, plus the methodical, inescapable WNV migration across the U.S. provided state and local public health officials ample opportunity to institute surveillance, diagnosis, public outreach, and control activities before WNV reached their borders.

## 5. Discovery of Human Mosquito-borne Encephalitis in New York City

It was into this patchwork of public health capabilities that WNV, an African flavivirus, entered the scene in New York City (NYC) in 1999 killing birds and humans [15]. The summer of 1999 was hot in NYC. Residents were sleeping outside, collecting water in containers for their plants and gardens, and permitting their swimming pools to lie dormant and become polluted. By mid-summer there were anecdotal reports of American crow (*Corvus brachyrhynchos*) deaths and “drunken crows” around the city. Unknown to federal officials at the time, some of the dead birds had been sent to the New York State Wildlife Pathology Laboratory for evaluation. There was an unusual summer outbreak of equine encephalitis in horses towards the eastern end of Long Island, later to be identified as WNV. Interestingly, illness and death in birds other than crows, e.g., Chilean flamingos (*Phoenicopterus chilensis*) and a snowy owl (*Bubo scandiacus*), occurred in the Bronx Zoo. The human outbreak did not come to the attention of CDC until Deborah Asnis, a local physician in Queens, reported a cluster of human encephalitis cases to the NYC Department of Health (NYCDOH) and the CDC was contacted to assist in a human outbreak investigation by Drs. Marcelle Layton and Annie Fine of the NYCDOH [16,17]. 

Human serum specimens were sent to the CDC’s Division of Vector-Borne Diseases (DVBD, Fort Collins, CO, USA) by the NYCDOH for serological evaluation. It was quickly determined that these human cases had only SLEV-reactive IgM in the acute-phase serum using screening MAC-ELISA on the North American arboviral antigen panel. There were no specimens available from which virus could be isolated and identified, so paired acute- and convalescent-phase human serum specimens were tested using the more specific PRNT. The PRNT results determined that all patients had a four-fold or greater rise in anti-SLEV virus-neutralizing antibodies. These antibodies did not neutralize EEEV, LACV, DENV, YFV, or POWV. The results fit a diagnosis of SLEV. 

Because of the MAC-ELISA and PRNT results, a team of CDC scientists were immediately dispatched to the NYC to assist in the outbreak investigation of the first SLEV-outbreak recorded in NYC. The public health response to the outbreak could not wait for confirmatory diagnostic results from virus isolation, because appropriate specimens were not available. Within a few days of the CDC serological identification of SLEV, the mayor of NYC instructed the NYCDOH to begin an aggressive, multi-faceted, public health intervention in the borough of Queens. This intervention ranged from distributing mosquito repellent at the U.S. Tennis Open to truck-based and aerial application of mosquito adulticides. After new cases were diagnosed in Brooklyn, Bronx, and Manhattan, vector-control intervention was enlarged to include the four contiguous boroughs of NYC, but not Staten Island, which did not register a WNV case until 2000.

Diagnostic work continued at the CDC and other federal facilities as reports of bird deaths and human infections accrued. A linkage of human and bird outbreaks would have been unusual for SLEV, since it had never been associated with bird fatalities. Furthermore, this observation was true for all flaviviruses that used birds as amplifying hosts—including WNV—which was why when this author solicited opinions from a variety of other arboviral experts like Robert Shope, Tom Monath, and Bob McLean, no link to WNV was made. By this time, however, animal tissue specimens had been disseminated to a variety of Federal animal labs—the DOD, USDA’s National Veterinary Services Laboratory (NVSL), and Dr. McLean’s National Wildlife Health Center (NWHC) labs in the DOI. No single lab identified the virus agent as WNV. This was in large part due to the limited testing capacities of each facility.

**Figure 3:**
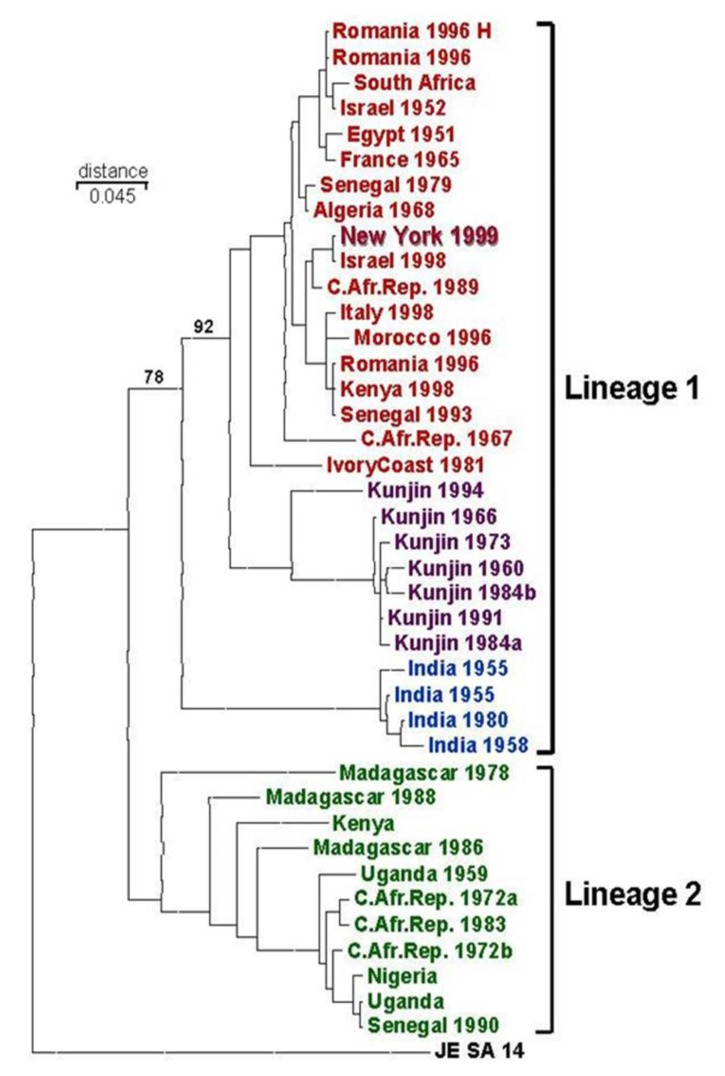
Phylogeny of WNV based on gene sequence of the envelope protein as of 1999 [21].

Unknown to the scientists at the CDC, DVBD, a meeting of the CDC-sponsored Unknown Encephalitis Project was occurring in Albany, NY, at the time of the NYC outbreak. The vast majority of human encephalitis goes undiagnosed. Because of this, CDC initiated the Unknown Encephalitis Project—supported by its Emerging Infections Program—to organize laboratory testing of specimens from undiagnosed human encephalitis cases. In this Project, specimens collected by state and local public health labs were forwarded to CDC where comprehensive diagnostic capabilities beyond those of the states existed. Specimens were logged in and then divided among CDC’s disease agent-specialty laboratories for diagnostic evaluation. At the Albany meeting specimens from the NYC human outbreak were provided to Dr. Ian Lipkin, University of California-Irvine, by NY State DOH (NYSDOH) virologists. Scientists in Lipkin’s laboratory used sequencing techniques to identify viral nucleic acid in human tissue. Examination of their published phylogenetic analysis of NS3 and NS5 gene sequences indicated that the Australian KUNV was the closest match with the NYC flavivirus [18]. As was noted previously, however, KUNV had never been associated with large outbreaks of human disease [19,20], and therefore they identified the virus Kunjin/West Nile-like virus. The data suggesting that KUNV was the culprit of the NYC outbreak was not surprising since both KUNV and the WNV from the NYC outbreak were Lineage 1 WNVs and were more closely related to each other than either were related to the Lineage 2 WNV genomic sequence available at that time (Figure 3). The identification of KUNV as the possible agent of the NYC outbreak caused confusion, however this study did suggest that a virus other than SLEV was involved in the human outbreak as work continued to confirm a diagnosis in both humans and birds. 

The confirmed diagnosis of WNV infection in both birds and humans was made by the CDC after obtaining avian specimens from the USDA NVSL [21]. Using electron microscopy, the NVSL found enveloped viruses “consistent with an alphavirus or flavivirus” in these specimens, and they forwarded the avian tissue to Dr. Rob Lanciotti at the CDC, DVBD. Dr. Lanciotti sequenced virus from these specimens and identified the avian virus as WNV (strain NY99). The link to humans was made in two ways. After WNV sequences had been identified, follow-up serology at the CDC DVBD showed that the SLEV-reactive antibody from infected humans was far more reactive with WNV than SLEV in both MAC-ELISA and PRNT—further implicating WNV in the human infections. Additionally, the CDC Infectious Disease Pathology Activity received human autopsy material from fatal NYC encephalitis cases, and identified WNV antigen in brain tissue using immunohistochemical techniques [22]. The CDC results further clarified the early CDC/UC-Irvine data, removing both SLEV and KUNV from consideration and confirming WNV as the cause of both the human epidemic and the avian epizootic. At the same time, identification of WNV was made from mosquitoes, crows, and a Cooper’s hawk (*Accipiter cooperii*) in Connecticut confirming that the regional outbreak was also due to WNV [23]. 

From a public health perspective, SLEV and WNV are very closely related mosquito-transmitted bird viruses, so the public health response to both is the same. While the evolution of diagnosis from SLEV to KUNV/WNV-like to WNV made for good copy in the popular NYC press, the final WNV identification was a formality. In fact, the practice of using geographic antigen panels to test for serologic evidence of viral activity worked as predicted. Because SLEV and WNV are closely related, the SLEV antigen screening detected the WNV antibodies in the serological assays. In the end, however, etiologic agent identification could only be confirmed by virus isolation and identification and gene sequencing [19,21,23]. The biology of WNV and SLEV in North America turned out to be very different, however. These differences, such as replication levels in vertebrate hosts, e.g*.*, American crows and house sparrows (*Passer domesticus*), permitted WNV to become established in the Western Hemisphere in a way very different from SLEV. 

## 6. Possible Origins of the 1999 WNV Outbreak

Contemporaneous to the identification of WNV, CDC investigations began attempts to determine the possible origins of WNV-NY99 and how it got to NYC. The last major outbreak of human WNV occurred in the mid-1990s in Romania, however smaller outbreaks in humans and equines had also been identified in other parts of the world between 1995 and 1999: Morocco (1996), Tunisia (1997), Italy (1998), and Israel (1998) [24,25,26]. Interestingly, as the North American outbreak progressed, a similar WNV outbreak was uncovered in Volgograd, Russia, at the same time [27,28,29,30]. 

Utilizing arboviral scientific networks it was learned that a fatal WNV outbreak in geese and storks had occurred in 1998 in Israel, however the findings had not been published [21,26,31,32,33]. The 1998 Israeli outbreak was the only outbreak to date where a flavivirus caused fatal bird infections—an observation consistent with WNV-NY99 [34]. To aid in the investigation, Vincent Deubel (Pasteur Institute, Paris, France) provided the E protein gene sequences from of a variety of WNV isolates—including the Israeli isolate. Roy Hall (University of Queensland, Brisbane, Australia) and Ernie Gould (Oxford University, Oxford, Great Britain) provided WNV-specific MAbs for epitope mapping studies [21,35,36,37]. Phylogenetic analysis of the E gene sequences by CDC indicated that the closest relative to WNV-NY99 virus was the Israeli 1998 WNV [21,38] (Figure 3). The genetic result implied that WNV-NY99 originated in Israel and migrated to NYC from there. This was an intriguing hypothesis made all the more interesting by the ability of both the Israeli WNV and WN99 to kill birds. 

The CDC Division of Quarantine (DQ) initiated a study to identify commerce between NYC and Israel. No specific conclusions were made, and this study was never published. It is conceivable that WNV-infected mosquitoes, eggs, or larvae could have been in “wet” shipments to the U.S. Other “wet” mosquito introductions have occurred before in the U.S. and other countries. The mosquito, *Ae. albopictus*, was introduced into the U.S. in wet used tires and into both the U.S. and the Netherlands in wet “lucky bamboo plant” shipments from Asia [39,40,41,42,43]. Vertical transmission of flaviviruses is not common; however it does occur with WNV [44,45,46]. It is now clear that WNV moves in birds. While there are migration routes from the Eastern to Western Hemispheres, it is more likely that if a bird brought WNV to NYC, it would have been an imported, infected bird. The virus could have been introduced in a WNV-infected human; however human viremia is not routinely high enough to infect mosquitoes. Infection of the native NYC-area mosquito population would have been necessary to cause the 1999 WNV outbreak. Without more information, it is unlikely we will ever know how WNV came to the U.S., nor where it came from. 

**Figure 4:**
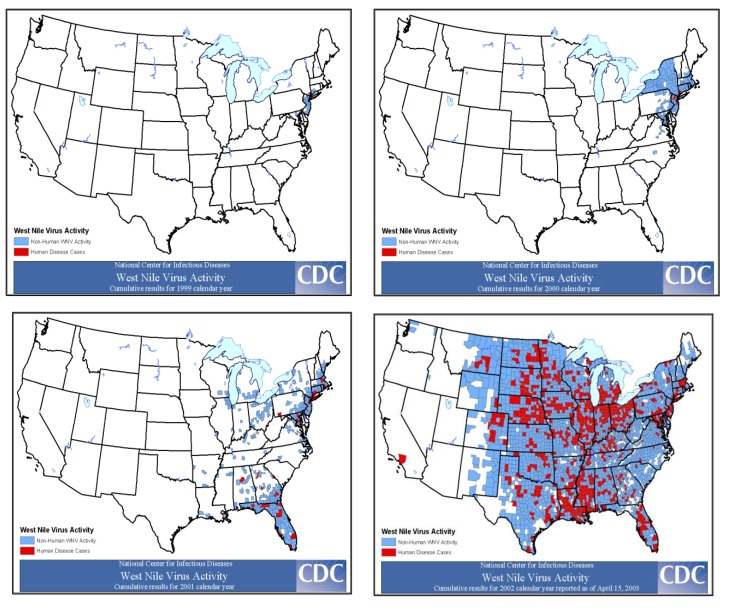
Yearly spread of WNV throughout the U.S. 1999–2002. Counties reporting WNV activity in humans (red) and non-human e.g., birds, mosquitoes, equines, and other mammals (blue), as reported to CDC ArboNet.

We will also never know precisely when WNV first entered the U.S. While it is presumed that this introduction occurred in the spring of 1999—immediately prior to the summer NYC outbreak—our experience derived from the subsequent spread of WNV throughout the U.S. suggests a different timing. As WNV moved across the U.S, the initial evidence of virus activity was found in infected mosquitoes or birds only—as WNV seeded itself into a new region. It was the year after the first evidence of WNV ecological activity that significant human outbreaks occurred. This pattern of activity suggests that WNV more likely entered the NYC area in 1998 or earlier (Figure 4).

## 7. Spread of WNV in the Western Hemisphere

**Figure 5:**
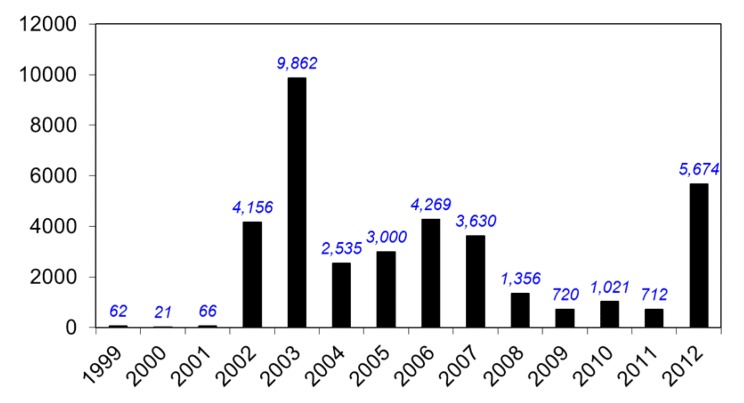
Yearly reported WNV human cases, 1999–2012, as reported to CDC ArboNet.

While the 1999 WNV outbreak in the NYC area was small—62 confirmed human cases—public health officials had no idea how extensive the outbreak really was, whether or not WNV could overwinter in the cold NYC area, or if it would spread beyond this geographic region. To answer these questions, three studies were performed in 1999 and 2000. The first study was a laboratory-based door-to-door serosurvey in Queens performed by the NYCDOH and CDC, to deduce the seroprevalence of WNV human infection in 1999 [47]. The result of the seroprevalence study indicated that the incidence of WNV human infection was 2.6% in the Queens’ study area (Table 2). Interestingly, a number of similar serosurveys have been completed since then, all demonstrating lower incidence results [48,49] (Table 2). Even though the number of human cases in the 1999 outbreak was small, the Queens serosurvey results and the corresponding ecological markers of WNV activity in birds and mosquitoes suggested an intense outbreak. As the number of human WN cases decreased from 2007–2011, it was hypothesized that most of the human population was becoming exposed and immune to WNV infection (Figure 5), though with an overall infection rate of only 2.6% during intense WNV transmission, the chance of this happening is small [50]. The large spike of human WNV cases in 2012 should remind us of the intermittent and cyclical nature of flaviviral epidemics.

**Table 2:** Seroprevalence studies of human WNV infection.

Location	State	Year	Clinical Cases	Serosurvey Sample Size	Identified Infection Rate	Estimated Infections
NYC (Queens)	NY	1999	62	677	2.6%	8200
NYC (Staten Island)	NY	2000	10	871	0.46%	1574
Suffolk County	NY	2000	0	834	0.12%	121
Fairfield County	CT	2000	1	731	0%	0

In the second study, collections of overwintering mosquitoes were obtained from protected areas like sewers, subways, *etc.*, in NYC. WNV-infected overwintering mosquitoes were obtained from the bunkers of Fort Totten, Queens [51]. This result suggested that indeed WNV would survive the winter and likely spread beyond the NYC area.

In the summer of 2000 residents of Staten Island, the fifth and final borough of NYC, fell victim to WNV, however there were only 21 confirmed human WNV cases in 2000. CDC designed a “transect” study to measure WNV dissemination from the immediate NYC area. Mosquitoes were collected along regularly placed transects radiating from the NYC area, and then were analyzed for the presence of WNV. By the time the study commenced in earnest, WNV could be detected in the outermost transect and the data were never published. These results predicted that WNV would indeed spread, and it would do so rapidly. By the end of 2000, WNV was detected as far south as North Carolina (Figure 4). In 2001 WNV continued its southerly march—presumably through bird migration—into Florida and other southern states. By 2002 WNV began to move west across the continent and into Canada. The largest WNV annual epidemic occurred in 2003 (9,862 reported human cases) as the virus reached the traditional range of SLEV, encountering a new, efficient mosquito vector, *Culex (Cx.) tarsalis—*plentiful in the irrigated farmlands of the Midwest and front range of the Rocky Mountains. This new mosquito vector had a longer flight range than the *Cx. pipiens* vectors of the east. Entry into the *Cx. tarsalis* vector population in addition to *Cx. pipiens* further hastened the dissemination of WNV across the western U.S. Currently, WNV can be found throughout the continental U.S. and also in Canada, Mexico, the Caribbean, and Central and South America.

**Figure 6:**
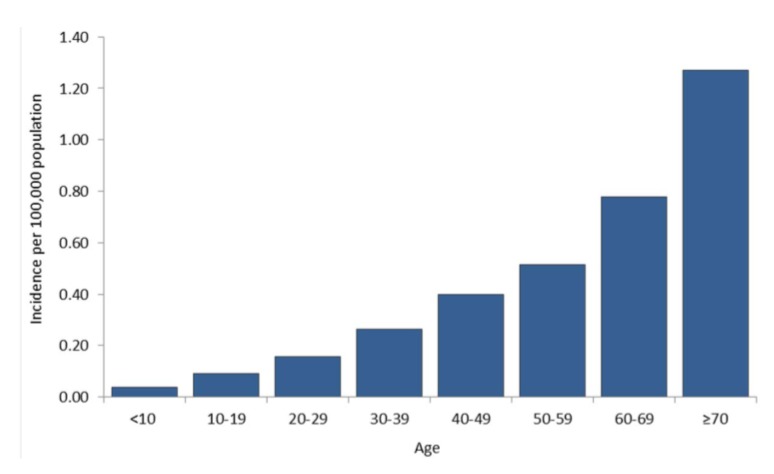
Average annual incidence of West Nile virus human neuroinvasive disease by age group, 1999–2012, as reported to CDC ArboNet.

**Figure 7:**
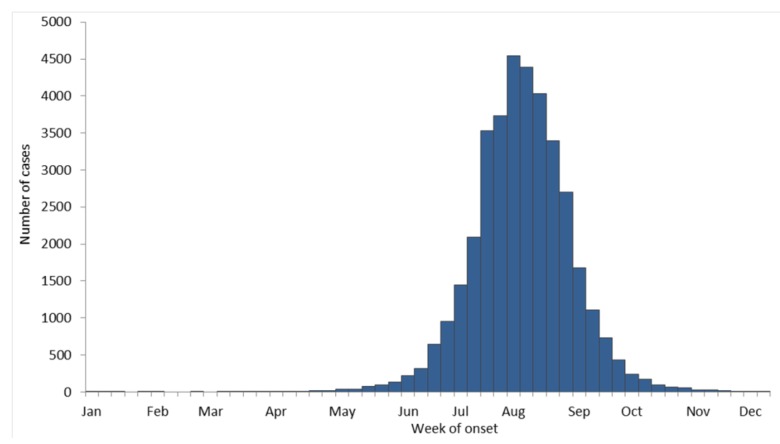
West Nile virus human disease cases reported to CDC by week of illness onset, 1999–2012, as reported to CDC ArboNet.

Even though WNV was new to North America, its epidemiology turned out to be very similar to SLEV. About 80% of human infections are silent. Through 2012 there have been 1,549 deaths due to WNV infection for a 4% case-fatality rate in clinical human infections. The disease is most severe in older people (Figure 6), however severe WNV disease can occur at any age, and “mild” WN is not necessarily mild. As with SLEV, transmission to humans begins in late spring and typically ends in the fall (Figure 7). The WNV transmission season can be longer in milder climates.

## 8. Federal Government Response to WNV

Because WNV was zoonotic the public and animal health response involved all of the before-mentioned federal agencies, and required the appointment of a WNV “czar” (Dr. Steven Ostroff, CDC) to coordinate the government’s response. Regardless of the broad spectrum of federal responders, there were some notable gaps in coverage, one of the most important being surveillance, detection, and identification of possibly zoonotic disease outbreaks in companion animals and in zoological parks. To be clear, even though the CDC received bird specimens from the NVSL, CDC was contacted during the outbreak by Tracy McNamara, a veterinary pathologist at the Bronx Zoo, requesting testing of specimens from unusual bird deaths at this zoo. Because this type of testing for bird diseases is usually performed at the USDA or NWHC, it was recommended that the specimens be submitted to these agencies for testing. The CDC does not maintain expertise in diagnosing most bird diseases—especially diseases occurring in exotic animals. In hindsight, it would have been more expedient for CDC to test directly the Bronx Zoo specimens rather than wait for them to arrive through the normal channels. As it turned out, no Federal agency had expressed responsibility to test specimens from zoos. This gap in coverage was later considered so significant that the CDC funded a Zoo Surveillance program at Cornell University’s School of Veterinary Medicine [52]. 

In the fall of 1999, Congressional and public health officials of the hardest hit states recognized the implications of the WNV outbreak and worked to secure additional CDC funding to assist the response efforts. In 2000, $2.7 million dollars was given to CDC to be distributed to affected states through its Enhanced Laboratory Capacity (ELC) cooperative agreement program. Virtually every state and some large municipalities like NYC had CDC ELC agreements, so this was a rapid mechanism for money distribution to help in preparing response plans as WNV progressed through the country. In subsequent years, the WNV-ELC funding increased to well over $20 million dollars. Human WN was made a nationally reportable disease, further facilitating surveillance efforts. A small amount of funding was used to jumpstart WNV research while the NIH geared up its infrastructure to develop a WNV research grant program. Another small amount of money funded university training grants to assist in increasing the national pool of trained arbovirologists. Vestiges of this funding remain today, but only to support lab diagnosis and WNV surveillance.

Besides engaging in diagnosis, surveillance, prevention and control responses, CDC hosted weekly national conference calls in 1999, with members of all states, some local jurisdictions, federal agencies, and Canadian public health officials. The conference calls were invaluable to public health officials as they responded to or prepared for WNV activity. Other federal agencies aided the WNV response. The USDA approved the first animal WNV vaccine. This killed virus vaccine abruptly reduced WNV infections in horses after implementation. The United States Geological Survey (USGS) collaborated with CDC to produce internet accessible, frequently updated county level maps of all states, tracking WNV human, equine, mosquito, and other animal infections. The FDA approved the first commercial diagnostic test for arboviruses, based on the CDC WNV MAC-ELISA. CDC scientists also developed a sensitive WNV-antigen detection ELISA [53]. This test was later adapted to a commercial lateral flow dipstick assay that permitted field identification of WNV-infected mosquitoes and dead birds [54]. A variety of WNV PCR based assays—some amenable to automation —were developed and deployed in state and local labs [55]. CDC began and still holds yearly diagnostic training classes in Ft. Collins to assure proper access to the latest diagnostic technology for international, state, and local public health laboratorians. In concert with this training, CDC conducts nationwide diagnostic proficiency testing of state diagnostic labs. Finally a CDC-developed WNV DNA vaccine was used to protect the entire population of California condors (*Gymnogyps californianus*) from WNV infection, thus saving the species from extinction [56,57,58]. To date, this is the only DNA vaccine to be approved by USDA and used in the open market.

There were other significant accomplishments made during this time. The first annual meeting on WNV in the U.S. was held in Fort Collins in the winter of 1999. Current stake-holders—federal, state, and local—were invited. Subsequent WNV meetings were held annually to provide public health officials with current information on WNV as it spread throughout the U.S. The CDC developed its first real-time disease reporting network—ArboNET—to track WNV disease in humans, equines, mosquitoes, and other mammals. States and local jursidictions receiving ELC funding were required to upload surveillance data to ArboNET on a weekly basis. These data were published weekly online and integrated into the online USGS maps. As an outgrowth of the WNV conference, CDC derived and published “West Nile virus in the United States: Guidelines for surveillance, prevention, and control.” These Guidelines have been recently updated (2013), but still serve as the source for all things related to a public health response to WNV. They are available on the CDC website. Finally in 2002, it was discovered that WNV could be transmitted between humans via blood transfusion or tissue transplantation [59,60,61,62,63,64,65,66,67,68]. This was the first time these modes of transmission had been identified for an arbovirus. The discovery led to universal screening of all blood donations in the U.S. using FDA-approved nucleic acid detection assays. Additional methods of human-to-human WNV transmission have also been identified, but they are very low incidence events [69].

## 9. Current Status of WNV and other Flaviviruses in the U.S.

**Figure 8:**
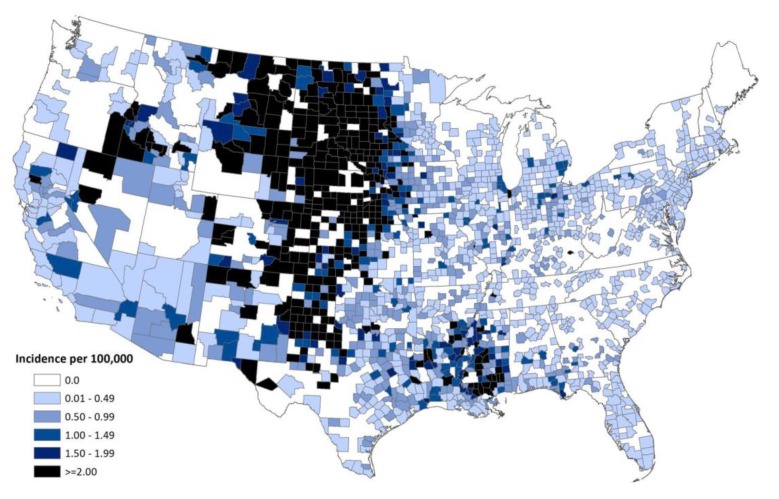
Average annual incidence of West Nile virus human neuroinvasive disease reported to CDC by county, 1999–2012, as reported to CDC ArboNet.

**Figure 9:**
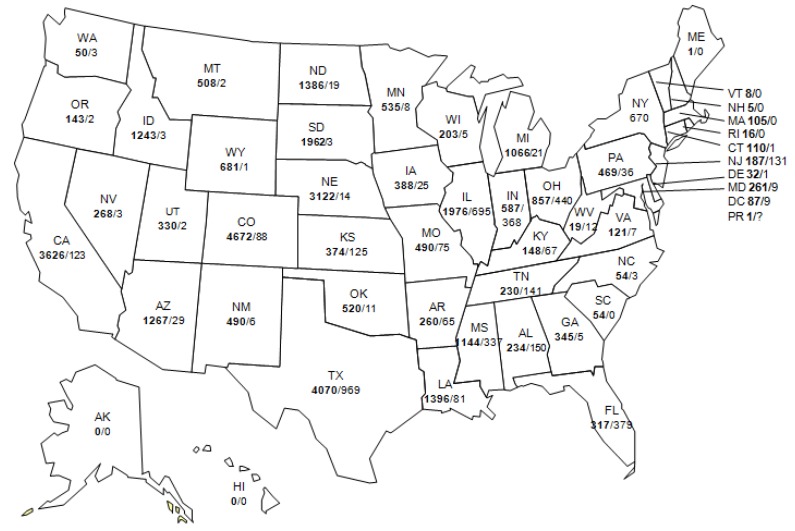
Total WNV and SLEV disease cases by U.S. state (WNV/SLEV). WNV, 1999–2012 (bold) cases reported to ArboNet. SLEV cases from 1964–2000 are listed [1].

**Figure 10:**
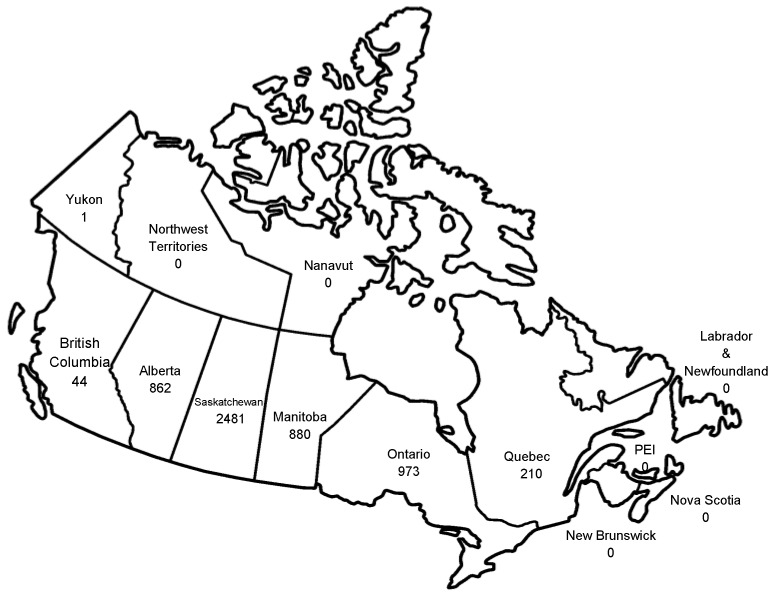
Total WNV disease cases by province, Canada, 1999–2012. Some cases assigned to a particular province were associated with travel outside of that province. Cases reported to the Public Health Agency of Canada [71]

WNV is now a permanent member of the arboviral “landscape” of the Western Hemisphere. Other Chapters in this special issue will deal in detail with other aspects of WNV. Since 1999 WNV has caused over 37,000 reported human cases (Figure 5), and is now the leading cause of mosquito-borne encephalitis in the U.S. and Canada (Figure 8, Figure 9 and Figure 10). As is the case with epidemic arboviruses, WNV activity ebbs and flows, but never really vanishes. After four consecutive years of low WNV activity in the U.S. (2008–2011) the over 5,000 cases in 2012 is the second most ever reported. Unlike the other endemic arboviruses in the U.S. that have very specific mosquito vectors and amplifying hosts, WNV is far less selective. It is likely that these characteristics alone will insure WNV infection of humans long into the future. Even though WNV and SLEV are very closely related (human SLEV infection can be just as devastating as human WNV infection) it appears that due to its “enhanced” biology, WNV might have actually displaced SLEV throughout much of the country. Over the years there have been a great many discoveries made in all aspects of the virus and its biology—too many to be delineated in this discussion. Many review articles and books have been written on the topic. The reader is encouraged to seek these writings out. For those who would like to have a more detailed—and fairly accurate account of 1999 WNV response—they are referred to a report from a U.S. General Accounting Office (GAO) investigation of it that is available online [70] 

## 10. Concluding Remarks

The first CDC WNV Guidelines included this priority research agenda for WNV:

- Determine current and future geographic distribution of WNV
- Study bird migration as a mechanism of WNV dispersal
- Study vector and vertebrate host relationships and range
- Identify and investigate virus persistence mechanisms
- Characterize mosquito biology, behavior, vector competence, surveillance, and control
- Develop and evaluate prevention strategies
- Improve laboratory diagnosis
- Determine the full clinical spectrum of disease and the long-term prognosis in humans
- Identify genetic relationships and the molecular basis of virulence
- Develop vaccines for animals and humans
- Develop antiviral therapy for WNV and other flaviviruses
- Determine the economic cost of the WNV epidemic/epizootic
- Investigate alternate modes of WNV transmission to humans

Thankfully many advances have been made in these areas and many discoveries have been implemented or documented in the scientific literature. WNV has fostered the careers of many scientists, some of whom are contributors to this special issue, and many of their discoveries are discussed in the following papers. 

In 2014 WNV will celebrate its 15th anniversary in the Western Hemisphere. It is safe to say that this event reshaped this generation’s perception of emerging diseases; however the WNV story is one that can be repeated by other pathogens. As with epidemic infections like SLEV in the 1960s, WNV has had its time in the public health spotlight. As a teenager in Central Illinois in the mid-1960s I remember the warnings about the mosquito-borne “sleeping” sickness. Everyone stayed indoors at night. It wasn’t until years later that I realized they were referring to SLEV. Unfortunately, in the shadow of public health policy and decreased public health funding, it’s the public that ultimately pays the price of epidemic arboviruses like WNV. 

Even though WNV is now an arboviral disease of the Western Hemisphere, new diseases like WNV can cause great disruption in advanced societies. It is a lesson that should not be forgotten. At the writing of this paper in 2013, the City of Fort Collins, CO, begins four days of staged adulticide spraying for WNV-infected mosquitoes. The Colorado cities of Loveland and Longmont have already completed their adulticide spraying. These current activities serve as a reminder that hard-earned progress in capacity building and infrastructure development for arboviruses—as was seen during the WNV epidemic—should not be easily relinquished. Unfortunately the public health community has suffered a loss of arboviral expertise and capacity. It is likely that the next introduction of an exotic arbovirus will again greet a woefully inadequate public health capacity beyond the federal level until a new rebuilding effort similar to what was accomplished in the early 2000s with WNV can be repeated. 
